# Diffuse intrinsic pontine glioma-like tumor with EZHIP expression and molecular features of PFA ependymoma

**DOI:** 10.1186/s40478-020-00905-w

**Published:** 2020-03-20

**Authors:** Drew Pratt, Martha Quezado, Zied Abdullaev, Debra Hawes, Fusheng Yang, Hugh J. L. Garton, Alexander R. Judkins, Rajen Mody, Arul Chinnaiyan, Kenneth Aldape, Carl Koschmann, Sriram Venneti

**Affiliations:** 1 Laboratory of Brain Tumor Metabolism and Epigenetics, Department of Pathology, University of Michigan, 3520E MSRB 1, 1150 W. Medical Center, Ann Arbor, MI 48109 USA; 2 Laboratory of Pathology, Center for Cancer Research, National Cancer Institute, Bethesda, MD USA; 3 Department of Pathology and Laboratory Medicine, Children’s Hospital Los Angeles, Keck School of Medicine University of Southern California, Los Angeles, CA USA; 4 Department of Neurosurgery, University of Michigan, Ann Arbor, MI USA; 5 Department of Pediatrics, University of Michigan School of Medicine, Ann Arbor, MI USA; 6 Michigan Center for Translational Pathology, Ann Arbor, MI USA; 7 Department of Pediatrics, Division of Pediatric Hematology/Oncology, C.S. Mott Children’s Hospital at the University of Michigan, 1500 E Medical Center Dr., Ann Arbor, MI 48109 USA

Diffuse brainstem gliomas, historically termed diffuse intrinsic pontine glioma (DIPG), account for approximately 75% of pediatric brainstem tumors and have a particularly poor prognosis with a median survival of only 10 months [8, 10]. Until recently, the diagnosis of DIPG was principally made by imaging, with biopsy relegated to an ancillary role owing to the delicate anatomic location [1]. However, with improved surgical techniques [4, 14] and the discovery of canonical histone H3 lysine-27-methionine (H3K27M) driver mutations, direct examination of these lesions to distinguish DIPG from radiologic mimics has reemerged as an important component of the diagnostic process [17, 19].

H3K27M mutations in DIPG result in global loss of the repressive H3K27 trimethylation (H3K27me3) through multiple mechanisms including inhibition of PRC2 methyltransferase activity and spread of H3K27me3 [5, 9, 18]. Global reduction in H3K27me3 is also observed in a subset of childhood posterior fossa (PF) ependymomas termed PF-group A ependymomas (PFA) [2, 12]. PFAs show overexpression of EZH inhibitory protein (EZHIP) in most cases, or harbor mutations in EZHIP in ~ 10% of tumors [11]. Additionally, three independent groups have demonstrated that EZHIP mimics the H3K27M “oncohistone” to cause global H3K27me3 reduction. EZHIP bears a methionine residue, similar to the H3 lysine-to-methionine (K27M) mutation, that is critical for mediating global H3K27me3 reduction [6, 7, 13]. The genomic distribution of H3K27me3 in H3K27M DIPGS and PFAs show remarkable similarities suggesting that these two tumors may be epigenetically related and share similar pathogenic mechanisms [2, 7]. Indeed, in support of this hypothesis, ~ 4% of PFAs demonstrate H3K27M mutations that are mutually exclusive from EZHIP mutations [11].

Here, we present an unusual case of a brainstem tumor with diagnostic radiographic and characteristic histopathologic features of DIPG, but demonstrating methylation features of PFA ependymoma. A 5-year-old boy presented to a local hospital with new-onset headache. Exam revealed evidence of cranial nerve deficits and cerebellar dysfunction (dysconjugate gaze and difficulty with tandem walking). MRI disclosed an infiltrating, expansile mass centered within the pons and obstructive hydrocephalus (Fig. 1a). The mass encased the basilar artery and contained a ventral exophytic component within the prepontine and suprasellar cisterns (Fig. 1b). Imaging characteristics included hyperintensity on T2 and fluid attenuated inversion recovery (FLAIR) sequences (Fig. 1c-d) and absence of enhancement following gadolinium administration (Fig. 1e). The mass did not involve the fourth ventricle or the cerebellum. Cystic change was noted. Other atypical features for DIPG, such as circumscription or dorsally exophytic growth, were not seen. The findings met clinicoradiologic criteria for DIPG [6]. Biopsy of the mass was deferred and radiation therapy was commenced.

**Fig. 1:**
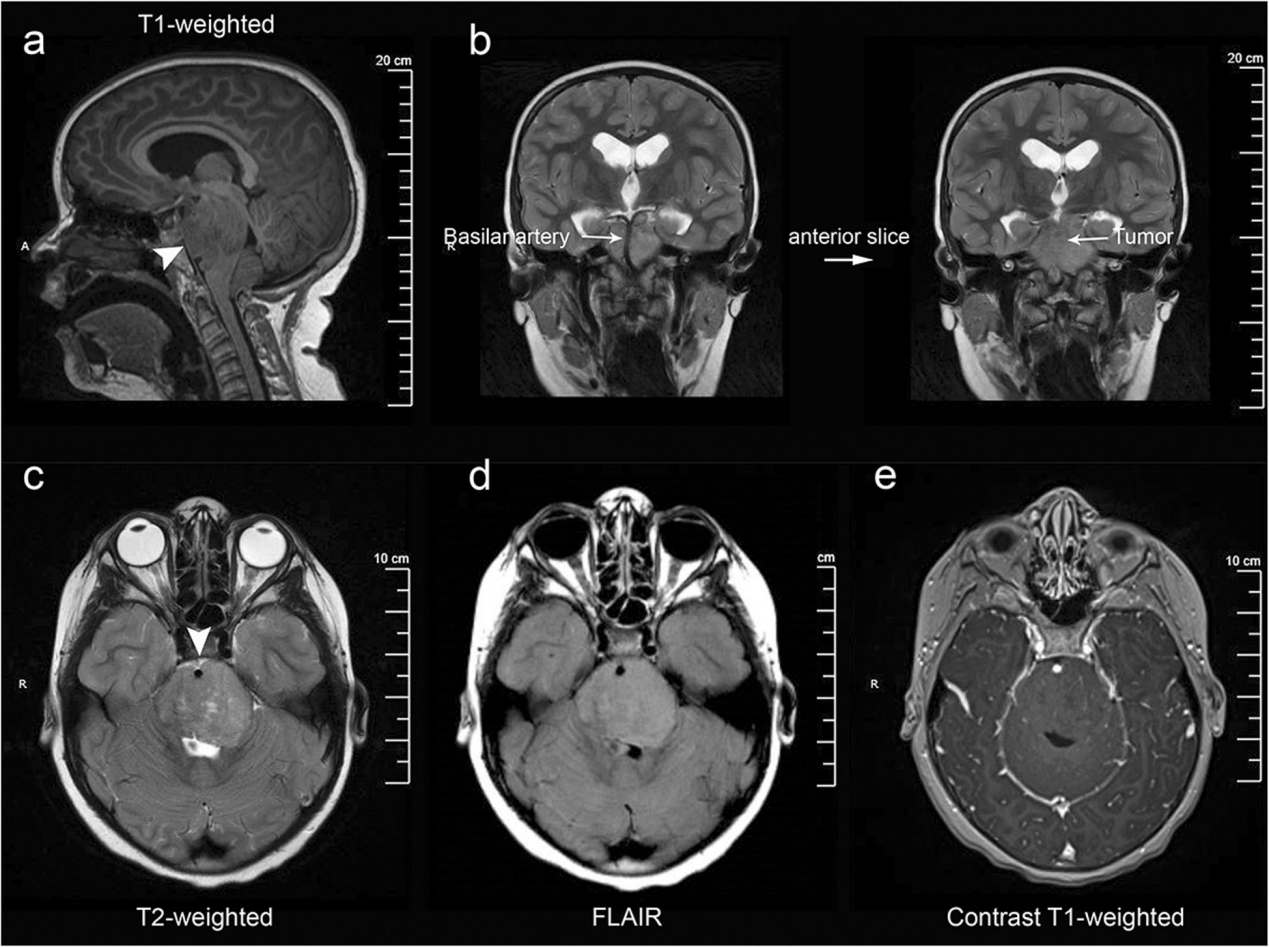
Representative MRI characteristics. T1-weighted sagittal image showed an infiltrative mass centered within and expanding the pons (**a**). The exophytic portion of the tumor is seen encasing the basilar artery (**b**). The mass demonstrated classic MR characteristics of DIPG including increased T2 (**c**) and FLAIR (**d**) signals, and lack of post contrast enhancement (**e**)

The patient was then evaluated at our institution for clinical eligibility in a trial for DIPG that prompted a tissue diagnosis. Biopsy revealed small fragments of densely cellular tumor (Fig. 2a). Scattered entrapped neurons hinted at its infiltrative nature (Supplemental Figure 1a-b). Tumor cells were positive for GFAP and neurofilament highlighted variable patterns of infiltration (Fig. 2b, Supplemental Figure 1c), with involvement of seemingly normal brain parenchyma by single tumor cells (Supplemental Figure 1e-f). No necrosis, microvascular proliferation, true ependymal or perivascular pseudorosettes were noted. Staining for H3K27M mutant protein was negative (Fig. 2c). Initial histologic and immunophenotypic findings suggested an H3-wildtype infiltrating astrocytoma consistent with DIPG. At our institution, pediatric CNS tumors frequently undergo integrative sequencing through the Michigan Oncology Sequencing Project (MI-ONCOSEQ) [16]. Tumors are assayed using whole exome and transcriptome-based techniques (see [15] for a description of the project). Sequencing revealed relatively few genomic alterations (Supplemental Table 1), but was notable for 1q gain (Fig. 2e) and confirmation of its H3-wildtype (*H3F3A*, *HIST1H3B*/*C*, *HIST2H3A*) status. RNA-seq showed overexpression of EZHIP mRNA that was confirmed by immunohistochemistry (Fig. 2f, Supplemental Figure 1d). Subsequent immunohistochemistry for H3K27me3 and Olig2 showed complete loss of nuclear expression in tumor cells (Fig. 2d, f). The relatively few genomic alterations and EZHIP overexpression in conjunction with loss of H3K27me3 in the absence of H3 mutations did not fit with classic molecular features of a H3-wildtype DIPG and prompted us to perform methylation analyses.

**Fig. 2:**
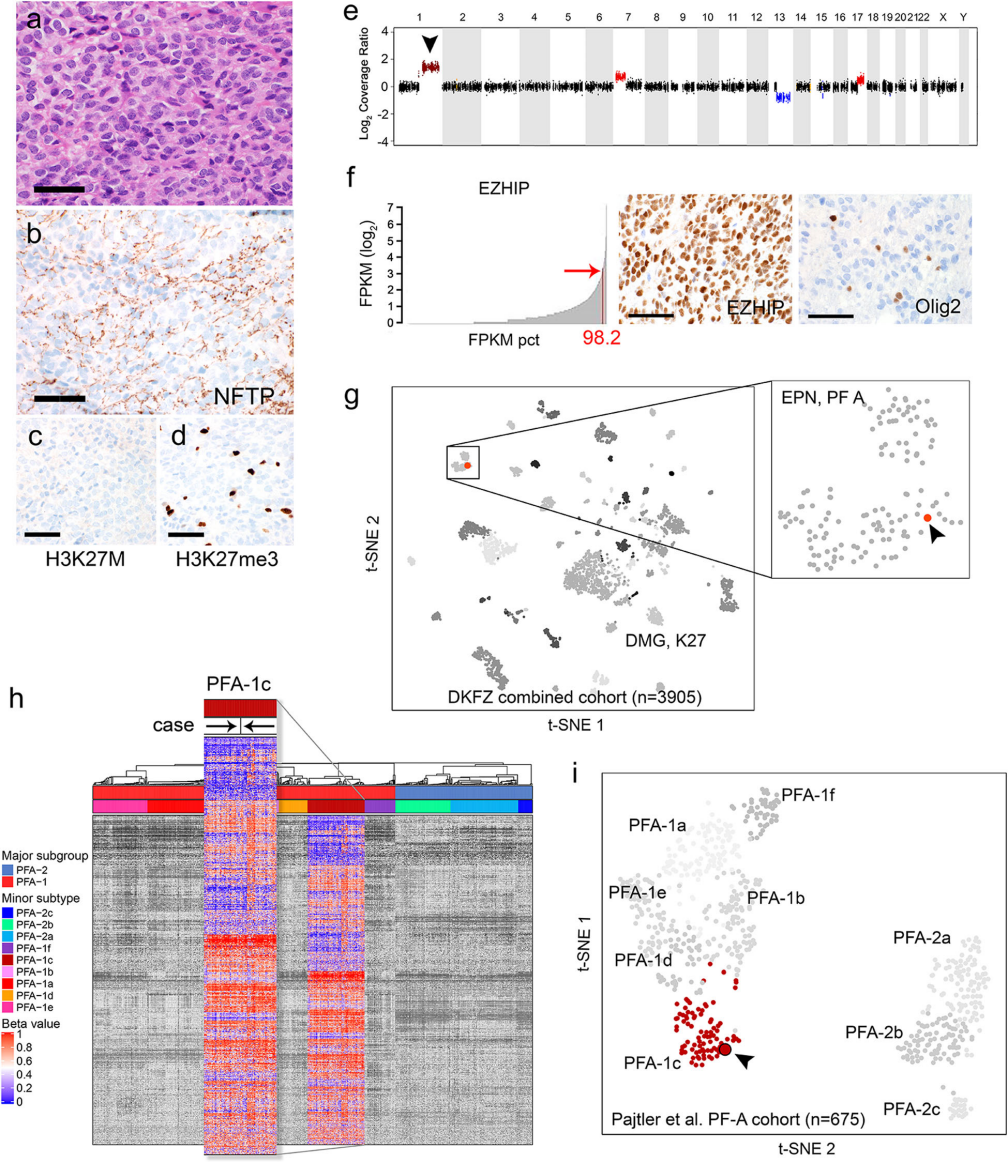
Histopathology and results of integrative sequencing and array-based methylation profiling. Routine H&E sections showed a high-grade cellular tumor (**a**). Tumor cells were seen infiltrating axons (**b**). H3K27M stain was negative (with appropriate control staining) and H3K27me3 showed loss with expression in admixed non-neoplastic cells (**c**, **d**). Copy number profiling through MI-ONCOSEQ mainly showed whole-arm changes including gains in 1q, 7p, 17p, and losses of 13q and partial 13p (**e**). Gene expression (RNA-seq) showed high expression of *EZHIP* (*CXorf67*) (f, expression is presented as log-transformed fragments per kilobase of exon model per million reads mapped, or FPKM, and shown as a percentile rank among the MI-ONCOSEQ compendium). Immunohistochemistry confirmed EZHIP protein overexpression and loss of Olig2 in tumor cells (**f**). Reproduction of the unsupervised clustering analysis of reference and diagnostic cohorts by Capper et al. [3] using t-Distributed Stochastic Neighbor Embedding (t-SNE), with incorporation of the presented case (**g**). Reproduction of the Consensus Clustering analysis by Pajtler et al. [11] and incorporation of our case showing clustering with the PFA-1c subtype (**h**). Heatmap representation and clustering were performed identically to the previously published methods [11]. Illustration of the previously defined [11] Consensus Clustering-based PFA major subgroups and minor subtypes (*n* = 675) using t-SNE dimensionality reduction (**i**); arrowhead denotes placement of the current case. Scale bars = 40 μm

Array-based profiling of CpG methylation in brain tumors has recently been shown to result in diagnostic refinements that are highly robust and prognostically meaningful [3]. We profiled our tumor using the Infinium MethylationEPIC BeadChip (interrogating ~ 850,000 CpG sites) in conjunction with the DKFZ Classifier tool recently implemented for CNS tumors (http://www.molecularneuropathology.org) [3]. While the methylation class most closely matched ‘ependymoma, posterior fossa group A’, the calibrated Classifier score was 0.62, below the proposed threshold of 0.9 (potential reasons are discussed below). To further assess the methylation profile of this tumor in relation to other CNS entities, we performed unsupervised clustering on the DKFZ cohort that comprises the 82 tumor methylation classes used in the Classifier (v11b4). Reproduction of the unsupervised clustering (t-SNE) demonstrated that the tumor clusters with the group ‘EPN, PF A’ (Fig. 2g). We next evaluated the tumor in relation to recently defined nine subtypes of PFA ependymoma [11]. Hierarchical clustering analysis revealed clustering within the PFA-1c subtype (Fig. 2h) and this was concordant with the results of t-SNE (Fig. 2i). While the overall Classifier score was 0.62, this may be due to the composition of PFA ependymomas in the current version of the Classifier (v11b4). The ‘EPN, PF A’ tumor class contains tumors arising solely within the fourth ventricle and/or cerebellum. Thus, the low calibrated score we encountered may reflect a potential subgroup of PFA ependymomas not yet recognized in the current implementation of the Classifier.

In summary, we present an unusual childhood brain tumor arising within the pons that met all clinical criteria for a DIPG but unexpectedly demonstrated H3K27me3 global reduction in the absence of H3.3 or H3.1 mutations, EZHIP overexpression, as well as clustering with tumors in the DNA methylation class ‘EPN, PF A’. While overall prognostic outcome has yet to be determined, a 7 month follow up MRI showed an increase in tumor size, portending an aggressive outcome. This case illustrates the significant contribution of molecular pathology to routine surgical neuropathology practice and highlights the increasingly important role of tissue procurement for genetic and epigenetic profiling of pediatric brain tumors in general. This case expands our knowledge of the epigenetic similarities including H3K27me3 global reduction between H3K27M DIPGs and PFA ependymomas [2, 7], and have biologic implications from both a neurodevelopmental perspective and in the design of targeted epigenetic therapies.

## Supplementary information

**Additional file 1: Supplemental Table 1.** Results of relevant alterations detected through MI-ONCOSEQ integrative sequencing.

**Additional file 2: Supplemental Figure 1.** H&E images showing entrapped neurons within the tumor mass (a-b). Neurofilament immunohistochemistry showing infiltrative densely cellular regions adjacent to more delineated areas (a). EZHIP (CXorf67) immunohistochemistry showed increased nuclear expression in tumor cells (d) and served to highlight individual tumor cells percolating surrounding normal-appearing brain (e-f). Scale bars = 40 μm (a-b), 100 μm (c-f).
