# Supplementary figures and images for: Diffuse intrinsic pontine glioma-like tumor with EZHIP expression and molecular features of PFA ependymoma
Source: Acta Neuropathol Commun. 2020 Mar 20;8:37. doi: 10.1186/s40478-020-00905-w (PMC7083001; doi:10.1186/s40478-020-00905-w)

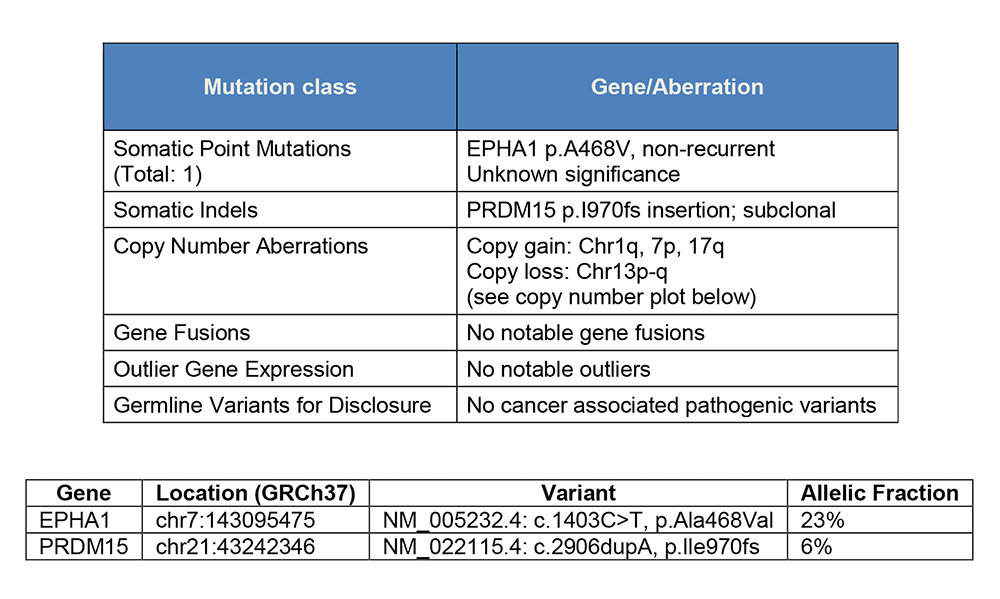

Supplement: Supplementary file 1 — Additional file 1: Supplemental Table 1. Results of relevant alterations detected through MI-ONCOSEQ integrative sequencing. [file 40478_2020_905_MOESM1_ESM.tif]

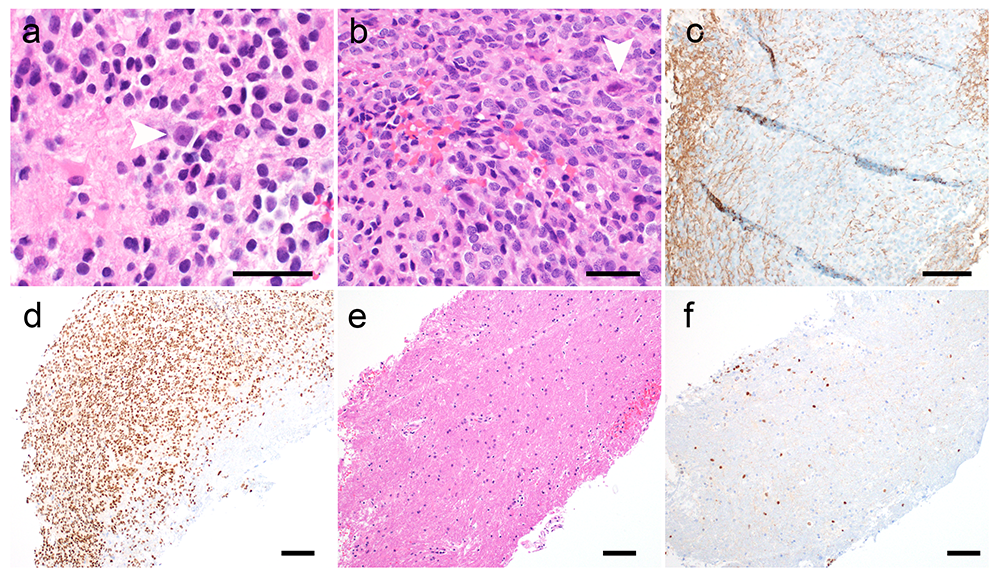

Supplement: Supplementary file 2 — Additional file 2: Supplemental Figure 1. H&E images showing entrapped neurons within the tumor mass (a-b). Neurofilament immunohistochemistry showing infiltrative densely cellular regions adjacent to more delineated areas (a). EZHIP (CXorf67) immunohistochemistry showed increased nuclear expression in tumor cells (d) and served to highlight individual tumor cells percolating surrounding normal-appearing brain (e-f). Scale bars = 40 μm (a-b), 100 μm (c-f). [file 40478_2020_905_MOESM2_ESM.tif]
